# Cost-effectiveness and value of information analysis of multiple frequency bioimpedance devices for fluid management in people with chronic kidney disease having dialysis

**DOI:** 10.1186/s12962-021-00276-6

**Published:** 2021-04-26

**Authors:** Elisabet Jacobsen, Moira Cruickshank, David Cooper, Angharad Marks, Miriam Brazzelli, Graham Scotland

**Affiliations:** 1 Health Economics Research Unit, University of Aberdeen, Polwarth Building, Foresterhill, Aberdeen, AB25 2ZD UK; 2 Health Services Research Unit, University of Aberdeen, Aberdeen, UK; 3 Chronic Disease Research Group, University of Aberdeen, Aberdeen, UK

**Keywords:** Multiple frequency bioimpedance devices, BCM—Body Composition Monitor, Cost-effectiveness, Value of information analysis

## Abstract

**Background:**

Among people with chronic kidney disease (CKD) on dialysis, sub-optimal fluid management has been linked with hospitalisation, cardiovascular complications and death. This study assessed the cost-effectiveness using multiple-frequency bioimpedance guided fluid management versus standard fluid management based on clinical judgment.

**Methods:**

A Markov model was developed to compare expected costs, outcomes and quality adjusted life years of the alternative management strategies. The relative effectiveness of the bioimpedance guided approach was informed by a systematic review of clinical trials, and focussed reviews were conducted to identify baseline event rates, costs and health state utility values for application in the model. The model was analysed probabilistically and a value of information (VOI) analysis was conducted to inform the value of conducting further research to reduce current uncertainties in the evidence base.

**Results:**

For the base-case analysis, the incremental cost-effectiveness ratio (ICER) for bioimpedance guided fluid management versus standard management was £16,536 per QALY gained. There was a 59% chance of the ICER being below £20,000 per QALY. Form the VOI analysis, the theoretical upper bound on the value of further research was £53 million. The value of further research was highest for parameters relating to the relative effectiveness of bioimpedance guided management on final health outcomes.

**Conclusions:**

Multiple frequency bioimpedance testing may offer a cost-effective approach to improve fluid management in patients with CKD on dialysis, but further research would be of value to reduce the current uncertainties.

**Supplementary Information:**

The online version contains supplementary material available at 10.1186/s12962-021-00276-6.

## Background

For people with chronic kidney disease (CKD) on dialysis, assessing hydration status and the amount of fluid to remove during a dialysis session are important clinical considerations. This has traditionally been a matter of clinical judgment, which can be unreliable leading to over or under-hydration and associated risks of hospitalisation, cardiovascular (CV) complications and death [1–9]. These complications contribute substantially to the high economic burden that CKD places on health systems [10]. Thus, scope exists to improve health outcomes and reduce costs to health services by improving fluid management decisions for dialysis patients.

In recent years, interest has grown in using multiple frequency bioimpedance devices to assess the hydration status of people on dialysis. These devices work by sending painless electrical currents through the body via electrodes, most commonly attached to the hand and foot [11]. Based on the impedance offered by different body tissues to different electrical frequencies, an algorithm is used to compute a person’s body composition (i.e. lean tissue, fat tissue, intracellular and extracellular water) [11–13]. In turn, this data can be used to estimate the amount of fluid that should be removed during dialysis in order to achieve normal levels of hydration.

In this study, we developed a health economic decision model to assess the cost-effectiveness of using a multiple frequency bioimpedance device to guide fluid management decisions in patients with CKD on dialysis, compared to the standard practice of relying on clinical judgment. The model was also used to identify gaps and sources of uncertainty in the existing evidence base, to help inform priorities for future research.

## Methods

### Model structure

A Markov model was developed in TreeAge Pro (TreeAge Software, Williamstown, MA, 2013). Full details of the model structure are published elsewhere [14].

**Fig. 1:**
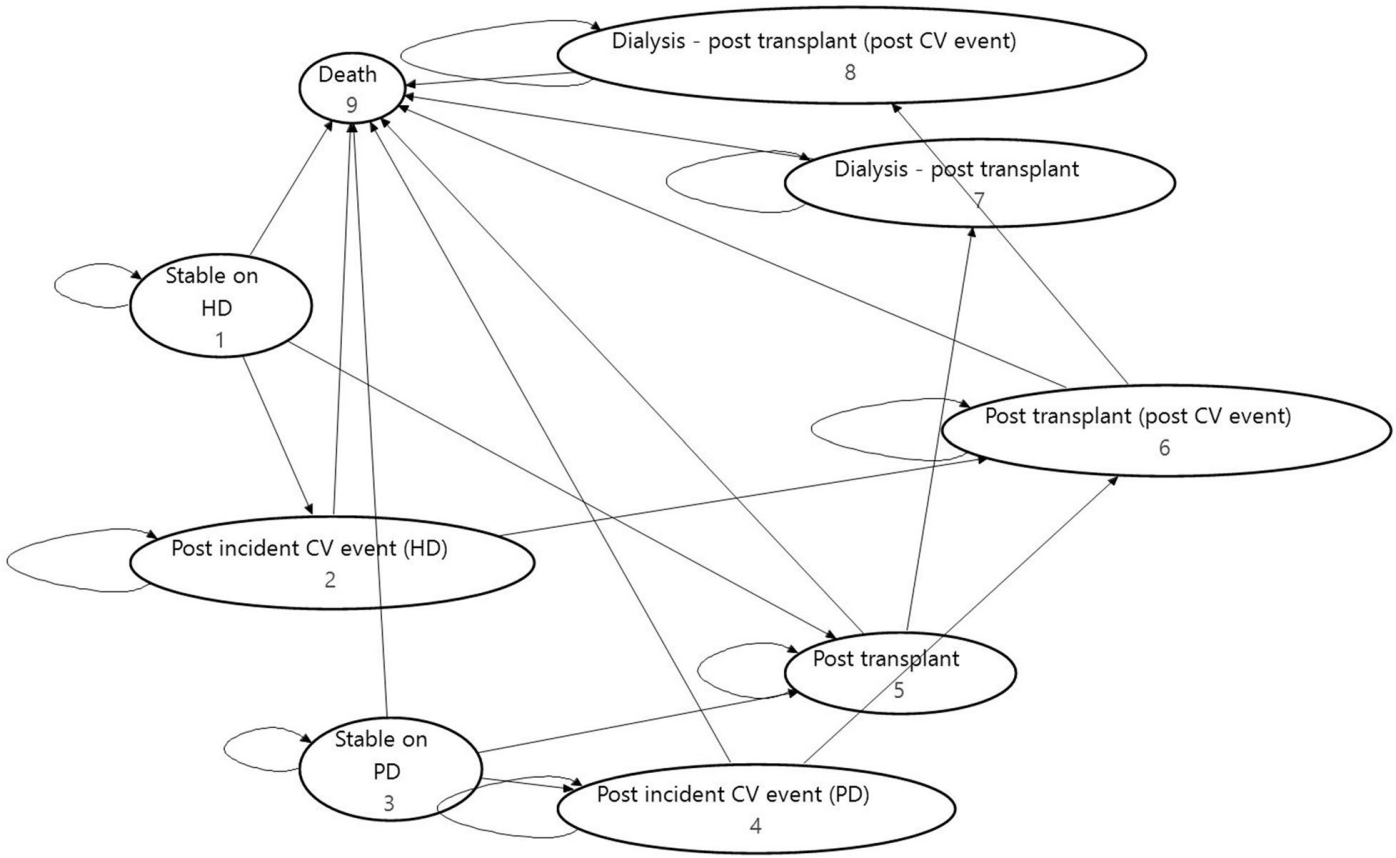
State transition diagram showing the baseline model structure

Briefly, the model simulated the flow of a mixed dialysis cohort (mean age 66 years, 61% male, 87% on haemodialysis (HD), 13% on peritoneal dialysis (PD)) through a set of discrete health states (Fig. 1). The distribution of the cohort across the model health states was updated on a fixed three-month cycle, based on a set of transition probabilities and clinical event rates derived from published literature. Costs and health state utility values were applied to the modelled health states and events, allowing cumulative costs and quality adjusted life years (QALYs) to be calculated for the alternative fluid management strategies over a 30-year time horizon.

### Clinical parameters

#### Baseline event risks

The baseline risk of mortality in the dialysis population was derived from the European Renal Association annual report [15]. A regression method was used to fit a Weibull function to the 5-year survival curve for a 60-year-old mixed dialysis cohort. The derived survival curve was adjusted to the starting age of the modelled cohort (66 years) using a hazard ratio for mortality associated with increasing age in the renal replacement therapy (RRT) population [1]. To minimise uncertainty associated with the use of parametric curves to extrapolate long-term survival, age specific relative risks for mortality in the RRT population [1] were applied to UK general population age/sex specific mortality rates [16] beyond 10 years in the model.

To estimate transitions from dialysis to renal transplant, we used the reported median time to transplant in the UK (1082 days) [17], applied to the proportion of the prevalent dialysis cohort waitlisted for transplant (13.5% for patients aged 65–75 years) [18]. No transplants were assumed to occur beyond the age of 75 in the model.

The ERA-EDTA Registry annual report [15] was used to inform post-transplant survival probabilities by type of donor (deceased/living) in the first year following transplant. Beyond year one we utilised 10-year Kaplan Meier data from a UK population-based study of transplant recipients [19]. The method reported by Hoyle et al. [20] was used to reconstruct individual patient survival data for 2887 subjects aged 60–69 years, before fitting parametric survival curves using R statistical software [21]. A Weibull function was chosen for application in the model based on the Bayesian information criterion and was further adjusted to the recipient’s age at time of transplant [19]. Beyond ten years, mortality in the post-transplant states was estimated by applying an adjusted relative risk [22] to age specific UK general population mortality rates.

Probabilities of unplanned hospitalisation were included in the model based on the first part of a published two-part cost model [10] developed to predict annual inpatient hospitalisation costs in UK dialysis patients. This allowed for prediction of annual hospitalisation risk by age, dialysis modality, time on dialysis, and the presence of various comorbidities. These predicted probabilities were transformed into 3-monthly health state specific probabilities of hospitalisation for application in the model. They were further disaggregated into CV (17.6%) and other causes (82.4%) using data reported by Rayner et al. [23]. Incident CV hospitalisations were assumed to result in an increased comorbidity burden, increasing the probability of subsequent hospitalisations from the post-CV event health states.

#### Clinical effectiveness

A systematic review was conducted to inform the clinical effectiveness of multiple frequency bioimpedance guided fluid management versus standard fluid management for people with CKD on dialysis. The review, reported in detail elsewhere, [14] identified five relevant randomised controlled trials (RCTs) [24–29]. The length of follow-up ranged from 3 months to 2.5 years. The total number of randomised participants was 939.

The identified trials were powered primarily on surrogate endpoints rather than final health outcomes. In a meta-analysis, both absolute overhydration and relative overhydration were found to be significantly lower in patients evaluated using BCM measurements to guide fluid management than for those evaluated using standard clinical methods [weighted mean difference − 0.44, 95% confidence interval (CI) − 0.72 to − 0.15, p = 0.003, I^2^ = 49%; and weighted mean difference − 1.84, 95% CI − 3.65 to − 0.03; p = 0.05, I^2^ = 52%, respectively]. Pooled effects on systolic blood pressure (SBP) (mean difference − 2.46 mmHg, 95% CI − 5.07 to 0.15 mmHg; p = 0.06, I^2^ = 0%), and arterial stiffness (mean difference − 1.18 m/s, 95% CI − 3.14 to 0.78 m/s; p = 0.24, I^2^ = 92%), whilst not statistically significant, directionally favoured bioimpedance guided fluid management.

The mean reduction in arterial stiffness was considered the most robust predictor of plausible effects of bioimpedance guided fluid management on non-fatal CV events and all-cause mortality in a dialysis population. Therefore, the mean reduction (measured as pulse wave velocity (PWV)) was combined with hazard ratios from a published observational study describing the relationship between PWV and a composite outcome of all-cause mortality and non-fatal CV events [30]. Verbeke et al. [30] showed that the relative effect per unit decrease in PWV in the dialysis population also decreased across tertiles of aortic calcification. The weighted average of the reported hazard ratios (by aortic calcification tertiles) was therefore calculated (0.942, 95% CI 0.879–1.009) and then scaled in the model to the pooled mean reduction in PWV (− 1.18, 95% CI − 3.14 to 0.78) observed for bioimpedance spectroscopy versus standard clinical assessment. The implementation of this approach used probabilistic sampling from distributions applied to both the pooled mean reduction in PWV, and the effect of a unit change in PWV. Therefore, the uncertainty surrounding both these inputs was propagated through the model, with the estimated composite effect of bioimpedance guided fluid management on all-cause mortality and non-fatal CV hospitalisation being 0.932 (95% CI 0.829–1.048).

### Health state utility values

A focused search was conducted to identify health state utility values (HSUV) in people with ESRD. HSUVs reflect the desirability of different health states on scale anchored by death (0) and full health (1), and can be combined with estimates of time (in years) spent in different health states to estimate QALYs.

Our search identified two previous systematic reviews which synthesised available HSUVs for modes of dialysis and transplant [31, 32]. The earlier meta-analysis [31] was preferred since it was restricted to studies reporting HSUVs based on the EQ-5D instrument. The reported pooled HSUVs were further adjusted relative to age related population norms in the model using a multiplicative approach [33]. In addition, published utility multipliers associated with CV events [33] were applied to patients experiencing incident CV events in the model. A utility decrement was also applied to hospitalisations for any other reasons, taken from an economic model developed to inform the National Institute for Health and Care Excellence (NICE) guideline on peritoneal dialysis [34]. Thus, the model was able to capture QALY gains associated with reductions in mortality, CV events and other cause hospitalisations.

### Costs

Direct health service costs included in the model were those for dialysis [35], kidney transplantation [35], post-transplant follow-up and immunosuppression [36–38], background medication (blood pressure medication [39, 37] and erythropoietin stimulating agents [18, 37]), all cause inpatient hospitalisation [10], and outpatient costs [10]. An additional cost of quarterly bioimpedance monitoring was applied to those in the bioimpedance arm of the model. All costs were expressed in 2014/2015 pounds sterling. Modelled costs and QALYs were discounted at 3.5%. Details on the applied costs are provided in Additional file 1.

The costs of all-cause inpatient hospitalisations were modelled based on age, type of dialysis, time on dialysis and comorbidity status using the cost model developed by Li et al. [10] The predicted hospitalisation costs were incorporated per inpatient admission and vary by health state and time varying characteristics of the modelled cohort. Annual outpatient costs for dialysis and transplant patients were also taken from Li et al. [10] and were divided into quarterly costs for application in the model.

The additional cost per patient of bioimpedance testing was calculated based on the device cost (BCM—Body Composition Monitor), maintenance, consumables (electrodes and patient cards), and staff time. The total resources required for quarterly testing of patients was informed by a brief questionnaire sent to clinical experts based at six UK centres using multiple-frequency bioimpedance testing [14]. The average annual cost per patient year for quarterly testing came to £101.41.

### Analysis

Whilst dialysis costs were included in the model, a decision was made to exclude them from the analysis since their inclusion would prohibit the ability of technologies that increase survival on dialysis to appear cost-effective at standard thresholds [40]. The model was analysed probabilistically using 1000 random draws from probability distributions assigned to each uncertain input parameter (Additional file 1). A cost-effectiveness scatter plot was used to present the uncertainty surrounding the modelled joint difference in costs and QALYs, and the net monetary benefit (NMB) framework was used to identify the proportion of model iterations favouring each strategy at increasing cost-effectiveness thresholds (λ):

$${\text{NMB }} = \, \lambda *{\text{Effect}} - {\text{Cost}}$$

Further analysis was conducted to determine the expected value of perfect information (EVPI), and perfect parameter information (EVPPI) [41]. The EVPI and EVPPI calculations establish a theoretical upper bound on the value of further research to reduce current uncertainties surrounding all the model input parameters and individual or specific groups of input parameters respectively. The value of information analysis was conducted using the Sheffield Accelerated Value of Information (SAVI) tool [41].

EVPI calculations give an estimate of value of further research per individual patient. To scale these to the population level, information is required about the size of the population that stands to benefit over the expected lifespan of the health technology in question. The population was set to the dialysis population in England and Wales in 2014 (27,804) [42, 43], and the useful lifespan of the bioimpedance technology was assumed to be 10 years.

## Results

### Cost effectiveness analysis (CEA)

**Fig. 2:**
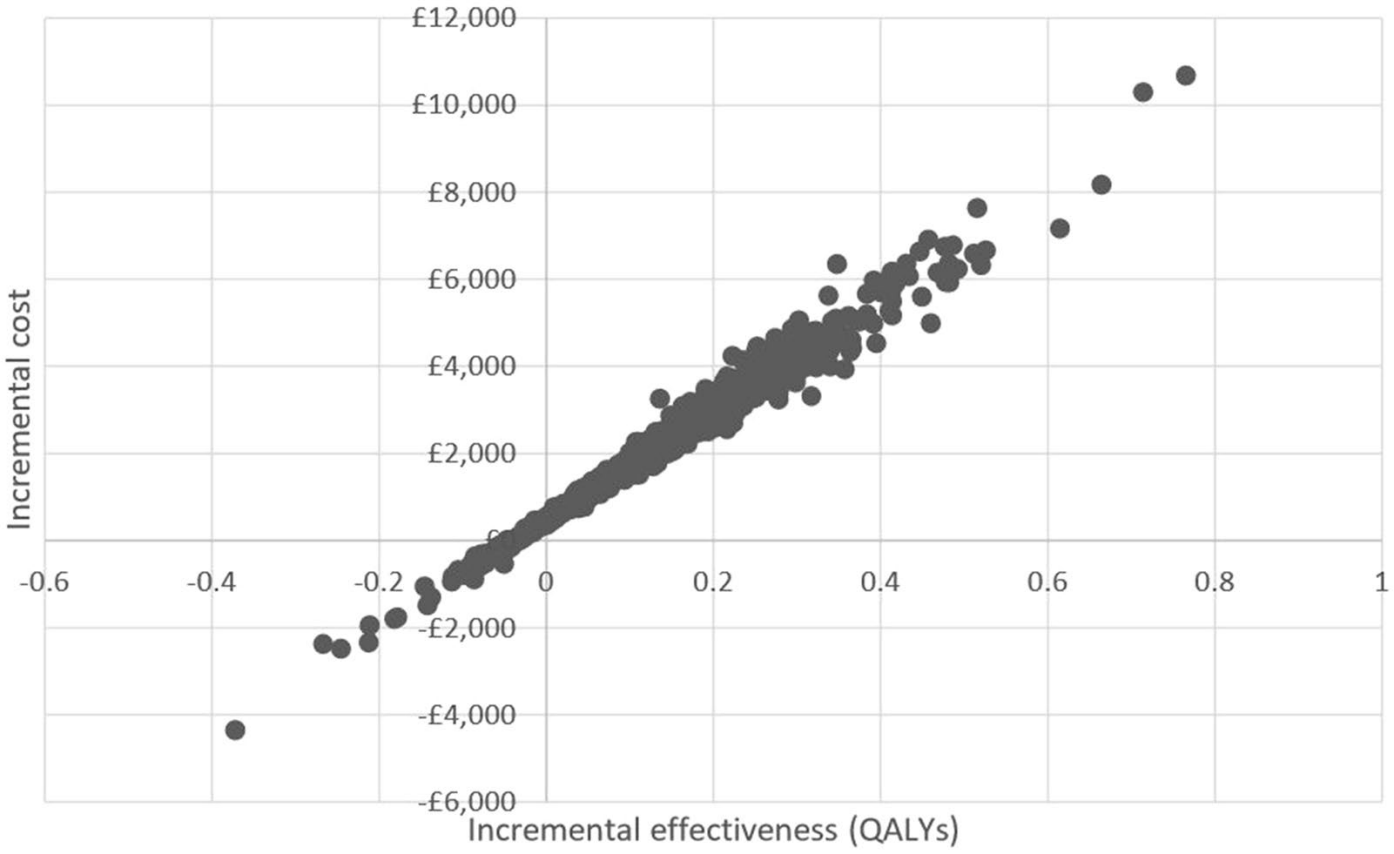
Incremental cost-effectiveness scatter plot: BCM—Body Composition Monitor versus standard care

**Fig. 3:**
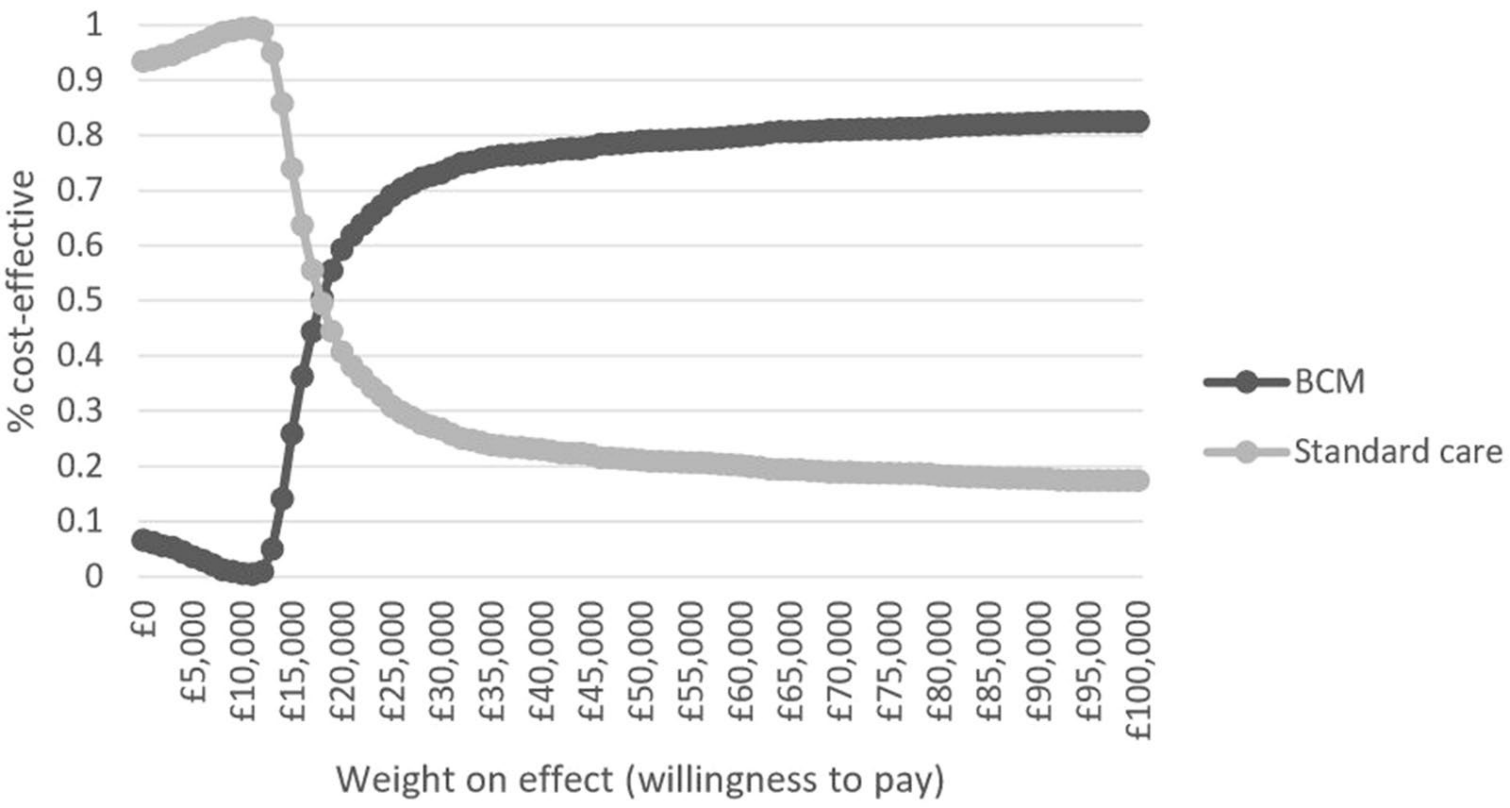
Cost-effectiveness acceptability curves: BCM—Body Composition Monitor versus standard care

The base case analysis showed that compared to clinical judgment alone, multiple-frequency bioimpedance guided fluid management resulted in increased costs to the health service of £1896 per patient for an average QALY gain of 0.115 over the 30-year time horizon (Table 1). The corresponding incremental cost-effectiveness ratio (ICER) was £16,536 per QALY gained. The probability of bioimpedance guided fluid management being cost-effective was 59% at the cost-effectiveness threshold of £20,000 per QALY gained. Figures 2 and 3 further summarise the uncertainty surrounding the cost-effectiveness findings. As the cost-effectiveness threshold (per QALY gained) increases, the probability of cost-effectiveness asymptotes to ~ 83%, the simulated probability that bioimpedance guided management would generate health benefits compared to standard fluid management.

**Table 1:** Base-case probabilistic cost-effectiveness scenario for bioimpedance guided fluid management versus standard practice (excluding dialysis costs)

Strategy	Mean costs	Incremental costs	Mean QALYs	Incremental QALYs	ICER	Probability cost-effective at £20,000 threshold
Clinical effectiveness: applying linked effects on mortality and non-fatal CV events through the pooled reduction in pulse wave velocity (HR = 0.9318 on both CV events and mortality)						
Standard care	£46,097		2.7031			0.407
BCM	£47,994	£1896	2.8177	0.1147	£16,536	0.593

### Sensitivity analysis

Scenario analyses were conducted to explore the impact of uncertainty surrounding several key input parameters (Table 2). Under most of the scenarios tested, the point estimate of the ICER remained below £20,000 per QALY gained. However, when excluding the effect on mortality, the ICER increased to £40,282.

**Table 2:** Deterministic cost-effectiveness scenario analyses for bioimpedance guided fluid management versus standard practice (excluding dialysis costs)

Strategy	Mean costs	Incremental costs	Mean QALYs	Incremental QALYs	ICER	Net monetary benefit (£)
Base case: applying linked effects on mortality and non-fatal CV events, estimated through the pooled reduction in pulse wave velocity (HR of 0.9318 applied to both all-cause mortality and CV hospitalisation)						
Standard care	£46,234		2.7014			7793
BCM	£48,153	£1919	2.8170	0.1157	£16,587	8188
1. Alternative to base-case clinical effectiveness scenario: applying the point estimate for the pooled effect of BCM on non-fatal CV hospitalisation events only (through the pooled reduction in PWV (HR = 0.9318 on CV events only)						
Standard care	£46,167		2.6976			7786
BCM	£46,391	£224	2.7032	0.0056	£40,282	7673
2. Alternative to base-case clinical effectiveness scenario: applying the point estimate for the pooled effect of BCM on mortality only (through the pooled reduction in PWV)						
Standard care	£46,234		2.7014			7793
BCM	£55,579	£9345	3.2719	0.5706	£16,378	9859
3. Apply a 10% reduction in the use of blood pressure medications						
Standard care	£46,234		2.7014			7793
BCM	£48,090	£1856	2.817	0.1157	£16,044	8250
4. Apply an increased cost of monitoring in adults by increasing the number of tests per patient to 12 annually (£229.65)						
Standard care	£46,234		2.7014			7793
BCM	£48,774	£2540	2.817	0.1157	£21,953	7567
5. Assume bioimpedance guided management results in a 2% improvement in the health state utility over the lifetime of dialysis patients						
Standard care	£46,234		2.7014			7793
BCM	£48,153	£1919	2.866	0.1646	£11,656	9166
6. Applying a smaller effect on mortality and non-fatal CV events (HR = 0.95 for both)						
Standard care	£46,234		2.7014			7793
BCM	£47,757	£1523	2.7853	0.084	£18,135	7949
7. Applying a larger effect of bioimpedance monitoring on both CV events and mortality (0.844); consistent with the cross-sectional main effect of a unit change in PWV reported by Verbeke et al. [30]						
Standard care	£46,234		2.7014			7793
BCM	£50,163	£3929	2.9791	0.2777	£14,145	9419
8. Excluding all non-CV event-related causes of hospitalisation from the analysis						
Standard care	£32,111		2.711			22,109
BCM	£33,412	£1,301	2.826	0.115	£11,311	23,108

### Value of information analysis

The EVPI for the decision between bioimpedance guided management and standard practice was £191 per patient and £53 million at the population level (Table 3). The EVPPI was highest (£187 per patient) for the parameter group determining the relative effect of bioimpedance guided fluid management on mortality and hospitalisation; i.e. the mean reduction in PWV and the hazard ratio per unit reduction in PWV. This suggests there would be value in conducting further trials powered to determine the clinical effectiveness of bioimpedance guided fluid management on these final health outcomes.

**Table 3:** Expected value of perfect information (EVPI) and perfect parameter information (EVPPI)

Parameter variables and groups	Per Person EVPPI (£)	Population EVPPI
Overall EVPI	£191	£53,160,000
Group EVPPIs		
Hazard ratio associated with a unit change in PWV	£187	£52,086,005
Mean change in PWV (m/s)		
Probability of graft failure with transplant from living donor	£1.31	£364,500
Dose of ESA in HD patients	£0.37	£104,000
Hazard ratio for mortality with transplant versus dialysis	£0.30	£84,200
Costs	0	0

## Discussion

### Principal findings

This study found that compared to standard care, bioimpedance guided fluid management is expected to result in increased costs to the health service of £1,896 per patient, for an increase in QALYs of 0.115. The corresponding incremental cost-effectiveness ratio is £16,536 per QALY gained. However, substantial uncertainty surrounds this point estimate, with there being a 41% chance that the ICER lies above the threshold of £20,000 per QALY.

The value of information analysis indicates a high value in further research to reduce the current decision uncertainty (EVPI = £53 million), with the highest value on parameters that drive the estimated effect of bioimpedance guided management on all-cause mortality and hospital admissions.

### Strengths and limitations

Key strengths of this study relate to the systematic approach used to develop and populate the economic model. The model structure itself was informed by a review of existing cost-effectiveness models in the area of CKD, and a systematic literature review of relevant RCTs was conducted to inform the clinical effectiveness inputs. Relevant registry data from the UK and Europe were also used to inform baseline mortality, all cause hospitalisation, and the probability of transplantation. This enhances the generalisability of the model findings to a prevalent, mixed dialysis population. In addition, a survey of clinical experts from centres experienced in using bioimpedance spectroscopy was undertaken to inform accurate costing of the testing pathway.

The key limitation relates to the availability of evidence to inform the effect of bioimpedance guided fluid management on final health outcomes. Identified trials of clinical effectiveness focused primarily on surrogate markers such as hydration status (as measured by bioimpedance spectroscopy), SBP, left ventricular mass index (LVMI), and arterial stiffness (PWV).

We explored evidence for linking changes in hydration status to all-cause mortality and hospitalisation rates, but there is a lack of clear reference standard for hydration status and further uncertainty relating to the nature and shape of the relationship. Most of the bioimpedance trials have focused on overhydration as an outcome [25–29], but other studies point to the avoidance of underhydration as potentially being equally important for the avoidance of vascular events [24, 44]. Furthermore, multiple fluid management parameters—including chronic volume expansion, ultrafiltration rate, and interdialytic weight gain—interact to affect CV morbidity and mortality [44]. For example, whilst overhydration in people on dialysis is associated with mortality, so too is a high ultrafiltration rate. There is potentially a risk to patients if overhydration is managed by more rapid ultrafiltration. Therefore, we focussed on more distal markers of vascular pathology to inform the modelling.

A systematic review of LVMI as a treatment target in ESRD was conducted in 2014 and concluded that there was no clear and consistent association between intervention-induced LVM change and all-cause or CV event-related mortality [45]. Heerspink et al. [46]. conducted a meta-analysis of RCTs evaluating blood pressure lowering medications in the dialysis population and estimated pooled relative risks of 0.71 (0.55 to 0.92) for CV events and 0.8 (0.66 to 0.96) for all-cause mortality, corresponding to a mean reduction in SBP of 4.5 mmHg. However, it is uncertain if reductions in SBP induced by blood pressure medication can be generalised to reductions in SBP induced by the management of fluid status. Therefore, a decision was made to model possible reductions in CV events and mortality through observed effects on arterial stiffness. Whilst there are limitations with this approach it serves to illustrate the potential for bioimpedance testing to be cost-effective (with modest effects on CV events and mortality) whilst acknowledging the uncertainty surrounding clinical effectiveness. Since all the clinical effectiveness evidence related to the BCM—Body Composition Monitor, generalisability of our findings to other multiple frequency bioimpedance devices is uncertain.

### Strengths and limitations with respect to other studies

To our knowledge, this is the first study to assess the cost-effectiveness of bioimpedance spectroscopy guided fluid management versus standard clinical management for the dialysis population. A review conducted by the Canadian Agency for Drugs and Technology in Health (~ CADTH) found insufficient evidence to support widespread adoption of the technology, highlighting a lack of cost-effectiveness evidence as one of the gaps in the existing evidence base.

### Meaning of the study

The model upon which the current analysis was based, was developed as part of Diagnostic Assessment Review commissioned by NICE in the UK [47]. Based on the assessment group report [48], the NICE appraisal committee concluded that there is currently not enough evidence (on final health outcomes) to recommend the routine adoption of multiple-frequency bioimpedance device monitoring to guide fluid management in people with CKD having dialysis in the NHS. Thus, instead the committee chose to support its use in research only, and encouraged centres already using the BCM—Body Composition Monitor to take part in further research and data collection. The results of our new value of information analysis support this conclusion.

### Recommendations for further research

Based on the value of information analysis reported here, it is clearly important that future research is designed to reduce the uncertainty surrounding longer-term effects on final health outcomes. Further research using multiple frequency bioimpedance testing to assess fluid levels in dialysis patients is currently ongoing. The UK BISTRO (BioImpedance Spectroscopy To maintain Renal Output Trial) trial [49] is evaluating the clinical and cost-effectiveness of bioimpedance spectroscopy compared to standard clinical management of fluid levels in incident haemodialysis patients with some residual renal function. The primary outcome is time to anuria, but data on hospitalisation, critical events (including CV events and deaths), and patient reported outcomes are also being assessed as secondary endpoints. This data will help inform cost-effectiveness in the specific population, but further trials to inform the value of multiple-frequency bioimpedance monitoring more widely in the prevalent dialysis population may also be warranted. Those centres that have already introduced routine multiple-frequency bioimpedance device measurement for dialysis patients may also consider conducting adjusted retrospective analyses to estimate effects on clinically relevant and intermediate outcomes before and after introduction.

Strengthening of the evidence regarding the interrelationships between fluid management parameters, more distal surrogate markers of vascular damage (e.g. fluid management-induced changes in blood pressure, arterial stiffness), and consequent changes in mortality and hospitalisation events, would also be beneficial. Ideally, data from relevant randomised studies should be used to quantify relationships between intervention-induced changes in the surrogate end-points and longer-term changes in health outcomes.

## Conclusion

In conclusion, the current evidence suggests that multiple-frequency bioimpedance device measurement may be cost-effective way of improving fluid management in patients with CKD on dialysis. However, substantial uncertainties remain that would benefit from further research.

## Supplementary Information

**Additional file 1.** Table with details on the model input parameters.

## Data Availability

Data used for the economic evaluation and value of information analysis was obtained from literature (see Reference list section). Further details on the clinical effectiveness review findings are available here: https://www.journalslibrary.nihr.ac.uk/hta/hta22010#/abstract.

## Abbreviations

CADTH: Canadian Agency for Drugs and Technology in Health
CKD: Chronic kidney disease
CV: Cardiovascular
ESRD: End stage renal disease
EVPI: Expected value of perfect information
EVPPI: Expected value of perfect parameter information
HD: Haemodialysis
HSUV: Health state utility values
ICER: Incremental cost-effectiveness ratio
LVMI: Left ventricular mass index
NICE: National Institute for Health and Care Excellence
NMB: Net monetary benefit
PD: Peritoneal dialysis
PWV: Pulse wave velocity
QALY: Quality adjusted life year
RCT: Randomised controlled trial
RRT: Renal replacement therapy
SAVI: Sheffield Accelerated Value of Information
SBP: Systolic blood pressure
VOI: Value of information
