# Supplementary material for: Cost-effectiveness and value of information analysis of multiple frequency bioimpedance devices for fluid management in people with chronic kidney disease having dialysis
Source: Cost Eff Resour Alloc. 2021 Apr 26;19:24. doi: 10.1186/s12962-021-00276-6 (PMC8077940; doi:10.1186/s12962-021-00276-6)
Supplement: Supplementary file 1 — Additional file 1. Table with details on the model input parameters. [file 12962_2021_276_MOESM1_ESM.docx]

**Additional file 1**

|  | **Value (95% CI)** | **Parameter distribution** | **Source** |
| --- | --- | --- | --- |
| **Starting age** | 66 | Applied deterministically | UK renal registry report, 2015(18) |
| **Gender (male) (%)** | 61 | Applied deterministically | UK renal registry report, 2015(18) |
| **Clinical parameters** |  |  |  |
| Proportion HD | 0.870 (0.865-0.873) |  | UK renal registry report, 2015 (18) |
| Mortality on dialysis (to 10 years) |  |  |  |
| *Weibull scale parameter (60 year old cohort)* | 0.114 |  | ERA-EDTA Registry annual report 2013 (15) |
| *Weibull shape parameter* | 1.035 |  | ERA-EDTA Registry annual report 2013 (15) |
| *Hazard ratio (10 year age increase on RRT)* | 1.65 (1.56-1.75) | Lognormal | UK Renal Registry Report(18) |
| Mortality year 1 post-transplant (deceased donor) |  |  | ERA-EDTA Registry annual report 2013 (15) |
| *Rate per patient year (0-19)* | 0.018 (0.006-0.029) | Lognormal |  |
| *Rate per patient year (20-44)* | 0.019 (0.016-0.022) | Lognormal |  |
| *Rate per patient year (45-64)* | 0.044 (0.040-0.048) | Lognormal |  |
| *Rate per patient year (65-74)* | 0.104 (0.090-0.120) | Lognormal |  |
| Mortality year 1 post-transplant (living donor) |  |  | ERA-EDTA Registry annual report 2013 (15) |
| *Rate per patient year (0-19)* | 0.007 (0.004-0.010) | Lognormal |  |
| *Rate per patient year (20-44)* | 0.007 (0.004-0.010) | Lognormal |  |
| *Rate per patient year (45-64)* | 0.02 (0.014-0.026) | Lognormal |  |
| *Rate per patient year (65-74)* | 0.053 (0.028-0.079) | Lognormal |  |
| Mortality post-transplant 1-10 years |  |  | Karim et al. 2014 (19) |
| *Weibull scale parameter (65 year old cohort)* | 0.05 | logscale multinormal |  |
| *Weibull shape parameter* | 1.027 | logscale multinormal |  |
| *Hazard ratio (10 year increase in transplant recipient age)* | 1.766 (1.540-2.028) | Lognormal | Karim et al. 2014 (19) |
| Hazard ratio for all-cause mortality with transplant versus dialysis (applied beyond ten years post-transplant) | 0.42 (0.16-0.76) | Lognormal | Tonelli et al. 2011 (22) |
| Proportion of prevalent dialysis population waitlisted for transplant |  |  | Annual report on Kidney Transplantation 2014; UKRRR, 2015 (17,18) |
| *<65 years* | 0.346 (0.338-0.354) | Beta |  |
| 65-75 years | 0.135 (0.128-0.142) | Beta |  |
| >75 years | 0 |  |  |
| Probability of transplant (3 monthly) among those waitlisted | 0.057 (0.055-0.058) | Beta | Annual report on Kidney Transplantation 2014 (17) |
| Probability of graft failure (3 monthly) |  |  | Annual report on Kidney Transplantation 2014 (17) |
| *Deceased donors* | 0.0075 (0.007-0.0081) | Beta |  |
| *Living donors* | 0.0047 (0.004-0.005) | Beta |  |
| *Proportion of transplants from deceased donors (age 60-70)* | 0.723 (0.706-0.40) | Beta | Karim et al. 2014 (19); Varies by age of recipient |
| Proportion of annual inpatient event rates that were CV events | 0.176 | 0.011 | Dialysis Outcomes and Practice Patterns Study (23) |
| **Treatment effect size** |  |  |  |
| Average effect per unit change in PWV across AAC tertiles (Hazard ratio) =1/(((1.154x1)+(1.154x0.895)+(1.154x0.865))/3) | 0.942  (0.879-1.009) | Lognormal | Assessment Group calculation based on Verbeke et al. (30) |
| Inferred average effect for a 1.18 reduction in PWV 0.942^1.18 (Hazard ratio) | 0.9318 (0.829-1.048) | Lognormal | Assessment Group calculation based on Verbeke et al. (30) |
| **Costs (£)** |  |  |  |
| HD per session | £154 (130-169) | Gamma | NHS reference costs, 2015 (35) Assumed 3 sessions per week. |
| PD per day | £69 (50-69) | Gamma | NHS reference costs, 2015 (35) Assumed one session per day. |
| Transplant | £14915 (11720-17797) | Gamma | NHS reference costs, 2015 (35) |
| Follow-up post-transplant (year 1) excluding immunosuppressant drugs | £11,204 | Applied deterministically | Treharne 2014 (36) |
| Immunosuppressant costs (year 1) | £10,622 | Applied deterministically | NICE GID-TAG348; expert opinion, BNF (38,49) |
| Annual immunosuppressant costs (beyond year 1) | £9,054 | Applied deterministically |  |
| Blood pressure medication for dialysis patients (Total average cost per year) | £88.76 | Applied deterministically | Tan et al. 2016 (UK study on proportions on different types of medication), BNF (Doses and prices) (37,38) |
|  | *Value (SE)* |  |  |
| Cost of erythropoietin stimulating agent for patients on dialysis (per year) |  |  | UK renal registry report, 2015(proportion of HD and PD on ESA and median dose (IU) per week), BNF (average price per IU for NeoRecormon and Aranesp) (18,38) |
| ESA dose (HD) | 7400 (2207) | Gamma |  |
| ESA dose (PD) | 4500 (1403) | Gamma |  |
| Proportion of HD population on ESA | 0.87 (0.079) | Beta |  |
| Proportion of PD population on ESA | 0.68 (0.112) | Beta |  |
| Price per IU (NeoRecormon) | 0.0070 | Applied deterministically |  |
| Price per IU (Aranesp) | 0.0073 | Applied deterministically |  |
| Estimated cost per year (HD) | £2,404 |  |  |
| Estimated cost per year (PD) | £1,142 |  |  |
| Estimated annual device cost (BCM Body Composition Monitor), including maintenance costs with parts and labour | £101.41 | Applied deterministically | Detailed calculations are available in Scotland et al. 2018. (14) |
| Hospital inpatient |  |  | Based on a published cost model by Li et al. Further details on costing methodology provided in Scotland et al. 2018 (10,14) |
| HD | ~£4500 |  | Variable predicted by cohort characteristics |
| PD | ~£4300 |  | Variable predicted by cohort characteristics |
| Hospital outpatient |  |  | Based on a published cost model by Li et al. (10) |
| Dialysis | £1312 (6.51) | Gamma |  |
| Transplant | £2606 (19.37) | Gamma |  |
| **Utilities** | *Age adjusted multiplier for use in model (SE)* |  |  |
| Stable HD | 0.678 (0.040) | Beta | Liem et al. 2008; Ara and Brazier, 2010 (31,33) |
| Stable PD | 0.694 (0.052) | Beta | Liem et al. 2008; Ara and Brazier, 2012 (31,33) |
| Stable post-transplant | 0.939 (0.053) | Beta | Liem et al. 2008; Ara and Brazier, 2013 (31,33) |
| MI within 12 months | 0.898 (0.056) | Beta | HSE data - Ara and Brazier 2010 (33) |
| MI history | 0.923 (0.025) | Beta | HSE data - Ara and Brazier 2010 (33) |
| Angina within 12 months | 0.782 (0.024) | Beta | HSE data - Ara and Brazier 2010 (33) |
| Angina history | 0.981 (0.019) | Beta | HSE data - Ara and Brazier 2010 (33) |
| Stroke with 12 months | 0.792 (0.048) | Beta | HSE data - Ara and Brazier 2010 (33) |
| Stroke history | 0.839 (0.023) | Beta | HSE data - Ara and Brazier 2010(33) |
| Any new CV event (within 12 months) | 0.832 (0.042) | Beta | Weighted average of parameters above |
| New CV event history | 0.931 (0.022) | Beta | Weighted average of parameters above |
